# Dysregulation of lncRNA–miRNA–mRNA Interactome as a Marker of Metastatic Process in Ovarian Cancer

**DOI:** 10.3390/biomedicines10040824

**Published:** 2022-03-31

**Authors:** Irina V. Pronina, Leonid A. Uroshlev, Alexey A. Moskovtsev, Danila M. Zaichenko, Elena A. Filippova, Marina V. Fridman, Alexey M. Burdennyy, Vitaly I. Loginov, Tatiana P. Kazubskaya, Nikolay E. Kushlinskii, Alexey A. Dmitriev, Eleonora A. Braga, Olga I. Brovkina

**Affiliations:** 1 Institute of General Pathology and Pathophysiology, 125315 Moscow, Russia; zolly_sten@mail.ru (I.V.P.); leoniduroshlev@gmail.com (L.A.U.); bioinf@mail.ru (A.A.M.); danilamihailovich@mail.ru (D.M.Z.); p.lenyxa@yandex.ru (E.A.F.); burdennyy@gmail.com (A.M.B.); loginov7w@gmail.com (V.I.L.); brov.olia@gmail.com (O.I.B.); 2 Vavilov Institute of General Genetics, Russian Academy of Sciences, 119991 Moscow, Russia; marina-free@mail.ru; 3 Pirogov Russian National Research Medical University, 117997 Moscow, Russia; 4 N. N. Blokhin National Medical Research Center of Oncology, 115478 Moscow, Russia; oncogen5@ronc.ru (T.P.K.); kne3108@gmail.com (N.E.K.); 5 Engelhardt Institute of Molecular Biology, Russian Academy of Sciences, 119991 Moscow, Russia; alex_245@mail.ru; 6 Federal Research and Clinical Center of Federal Medical-Biological Agency of Russia, 115682 Moscow, Russia

**Keywords:** ovarian cancer, primary tumors, differentially expressed genes, interactome, noncoding RNA, microRNA, lncRNA, mRNA, triplets

## Abstract

Ovarian cancer (OC) is one of the most common types of cancer among malignancies of the female reproductive system. This pathology is asymptomatic until advanced stages and has a poor prognosis. Our study aimed to search for lncRNA–miRNA–mRNA competing triplets that promote ovarian tumorigenesis. For this purpose, we analyzed tumor samples from the TCGA database and verified the results experimentally in a set of 46 paired samples of tumor and matched histologically unchanged ovarian tissues from OC patients. The list of RNAs selected in silico for experimental studies included 13 mRNAs, 10 lncRNAs, and 5 miRNAs related to epithelial–mesenchymal transition and angiogenesis. We evaluated the expression of these RNAs by qRT-PCR and assessed the correlation between levels of miRNAs, mRNAs, and lncRNAs. Sixteen significant triplets were revealed, in some of which, e.g., OIP5-AS1–miR-203a–c-MET and OIP5-AS1–miR-203a–ZEB2, both lncRNA and mRNA had sites for miR-203a direct binding. Transfection of the OVCAR-3 and SKOV-3 cell lines with the miR-203a mimic was used to confirm the novel links of miR-203a with ZEB2 and c-MET in OC. These connections suggest that the interactomes have the potential for diagnostics of metastasis at early onset.

## 1. Introduction

Ovarian cancer (OC) is a leading cause of death among gynecological cancers in women around the world. In Russia, the incidence of ovarian cancer is approximately 77 cases per 100 thousand population [1,2]. The five-year survival rate of patients with the first stage of the disease is 75%, with the second stage it is 41%, with the third stage 17%. OC is 10 times less common than breast cancer but 3 times more fatal [3]. The high mortality rate of this disease is explained by its latent development. As a result, 75% of women develop an advanced and widespread disease at the time of diagnosis [4,5]. Considering the growing number of OC cases, diagnostics at the early stages as well as initial stages of tumor metastasis is essential. Significant progress was made in this area with screening techniques including ultrasound, computed tomography, and magnetic resonance imaging [6,7]. However, in the case of small-sized malignant neoplasms, these methods are not effective. Therefore, the search for novel diagnostic methods that can help in OC identification and treatment is currently an urgent task. One such approach can be the detection of molecular tumor markers. This strategy is useful in several therapeutic stages: early detection of cancer development, monitoring of treatment dynamics, specification of metastasis, and prediction of possible progression.

In recent years, molecular alterations in cancer at an epigenetic level have been attracting more attention. Epigenetic regulation during tumorigenesis involves multiple steps, where DNA methylation and noncoding RNAs (ncRNAs) are the key players [8,9,10,11].

Indeed, in a previous study, we demonstrated that the aberrant methylation led to significant decrease in expression of 12 tumor-suppressive microRNAs (miRNAs): miR-124-3p, -125b-5p, -127-5p, -129-5p, -132-3p, -137, -148a-3p, -191-5p, -193a-5p, -203a, -339-3p, and -375 [12]. The results were obtained on a set of 76 paired (tumor/matched normal) ovarian samples. The mentioned above miRNAs exhibited differential expression in 59–76% of OC samples. Herewith, strong correlation was revealed between alterations in the promoter methylation status and the relative expression level for all 12 miRNAs. The hypermethylation of five miRNA genes (*MIR-124-2*, *-125B-1*, *-137*, *-203A*, and *-375*) showed statistically significant association with metastasis. Thereby, these miRNAs were additionally validated on a set of 13 primary tumors and matched peritoneal metastases. The obtained data evidenced that the miRNAs have potential as prediction biomarkers for OC dissemination.

In this study, we investigated related long noncoding RNAs (lncRNAs) and mRNAs and analyzed the shift in the lncRNA–miRNA–mRNA interactome. The fact is that noncoding RNAs such as miRNAs and lncRNAs affect the posttranscriptional level of protein-coding genes via the formation of competing endogenous RNA (ceRNA) complexes [13,14,15,16,17]. Moreover, the silencing of one element in this complex may drive expression explosion of the related elements [18,19]. Exploring such triplets in pathogenic conditions will contribute not only to diagnostic purposes but also to the basic knowledge of the mechanisms of OC pathology.

In the present study, we screened expression profiles of OC samples presented in the TCGA database. Genes that were differentially expressed (DE) in ovarian tumor samples compared to normal samples were identified. Next, correlations between levels of three types of RNAs in pairs miRNA–lncRNA, miRNA–mRNA, and lncRNA–mRNA were calculated. The significant triplets were studied experimentally. By qRT-PCR, we evaluated expression profiles of the in silico selected triplets in tumor samples and matched normal tissues from patients with OC and estimated correlations between levels of lncRNAs, miRNAs, and mRNAs indicating their crosstalk. Moreover, transfection of the OVCAR-3 and SKOV-3 cell lines was performed to confirm the interactions.

## 2. Results

### 2.1. In Silico Selection of lncRNAs and mRNAs

The TCGA-OV project includes transcriptomic data for 608 OC samples (https://portal.gdc.cancer.gov/projects/TCGA-OV, accessed on 27 May 2021). Among them, we selected 311 cases with primary tumors and without any treatment before biopsy sampling. Most patients (72%) in the selected subgroup had stage III OC with metastasis, which is consistent with the characteristics of our sample set (see Materials and Methods (Section 4)).

In total, 354 lncRNAs were DE (adjusted *p* (*p*.adj) < 0.05). The list of DE lncRNAs is presented in Supplementary Table S1. Upregulated DE lncRNAs (which comprised 45.6% of all DE lncRNAs) were used for further analysis.

There were 546 DE mRNAs (Supplementary Table S2), 48.4% of which were upregulated. These mRNAs were subjected to correlation analysis.

**Figure 1:**
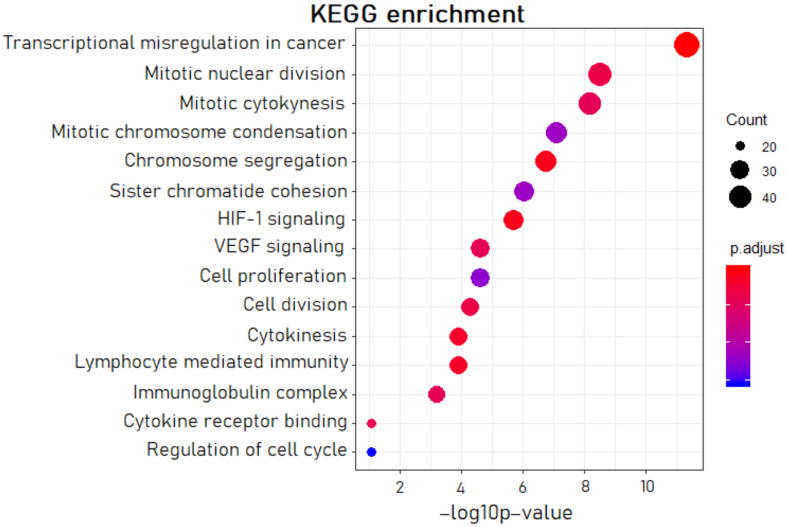
The most significant pathways in OC according to the gene set enrichment analysis (GSEA).

The gene set enrichment analysis (GSEA) confirmed the leading role of the transcriptional misregulation process in OC patients (Figure 1). The DE genes were also involved in cytokinesis, chromosome segregation, and other pathways combined with cell proliferation (altogether eight processes). We noted the group of four pathways referring to inflammation and angiogenesis: HIF-1 signaling, VEGF signaling, lymphocyte-mediated immunity, and cytokine receptor binding. The obtained GSEA data were consistent with the leading pathological processes in the tumor tissues of OC patients: growth and vascularization of malignant tumor, inflammation processes of abdominal organs, and hypoxia and acidosis associated with poor excretion of metabolites in a solid tumor.

The analysis revealed 102 statistically significant correlations between miRNA and DE lncRNA levels (Supplementary Table S3), while for miRNA–mRNA pairs, this number was higher and included 131 statistically significant direct or indirect interactions (Supplementary Table S4). Sixty-seven lncRNA–mRNA pairs showed positive or negative statistically significant Spearman’s correlation coefficients (Supplementary Table S5).

It appeared that the expression of many DE lncRNAs in the formed pairs was not detected in a part of the samples. Thus, we added a threshold for expression presence, which was set at >0 in more than 70% of samples. After this, we eliminated pairs in which the lncRNA expression did not meet this criterion. The same cutoff was applied for mRNAs.

As a result of this screening, we selected 13 mRNAs, 10 lncRNAs, and 5 miRNAs for experimental validation (Table 1). Of the selected RNAs, 46% were annotated as intracellular, 29% were extracellular, and 25% were both intra- and extracellular. We found no confidential information about the subcellular localization of MLK7-AS1 probably because this lncRNA is relatively novel among the selected lncRNAs.

### 2.2. Experimental Studies of the Level of Selected RNAs on Samples from OC Patients

**Figure 2:**
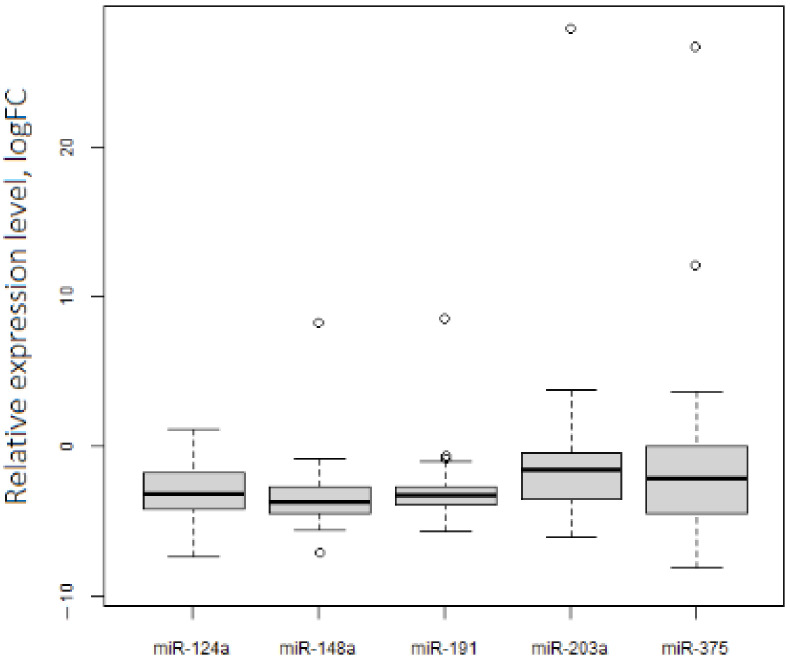
Expression levels of 5 tumor-suppressive miRNAs in OC. *p*.adj < 0.05 for each miRNA.

The qRT-PCR analysis of levels of 5 miRNAs in a set of 46 paired (tumor/normal) ovarian tissue samples showed their downregulation in OC (Table 1, Figure 2). However, we observed several outlying points with hyperexpression. Remarkably, 80% of these points were related to stage I OC cases.

**Figure 3:**
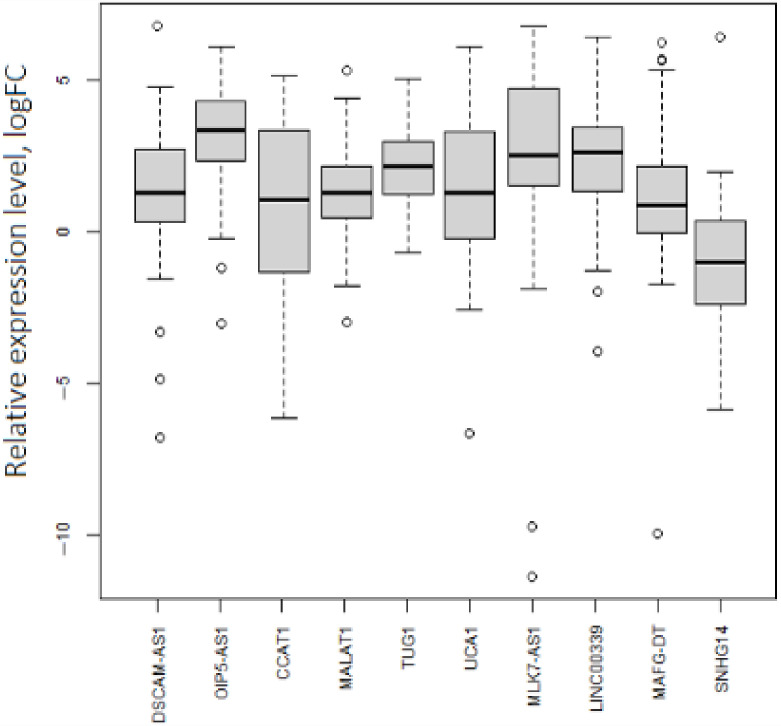
Expression levels of 10 lncRNAs in OC. *p*.adj < 0.05 for each lncRNA.

Not all experimentally obtained values of lncRNA expression in our sample set were consistent with ones in the TCGA-OV (Table 1, Figure 3). Namely, SNHG14 was downregulated in the analyzed by qPCR set (logFC (binary logarithm of expression level fold change (tumor vs. normal)) = −1.1). The outlying points with low expression levels were related to both the initial and late stages of OC. Expression levels of DSCAM1-AS1, CCAT1, MALAT1, MAFG-DT, and UCA1 were increased in ovarian tumors but were not markedly different from the normal tissue group. Nevertheless, OIP5-AS1, TUG1, MLK7-AS1, and LINC00339 had a perceptible expression increase in tumors (logFC ≥ 1.5).

**Figure 4:**
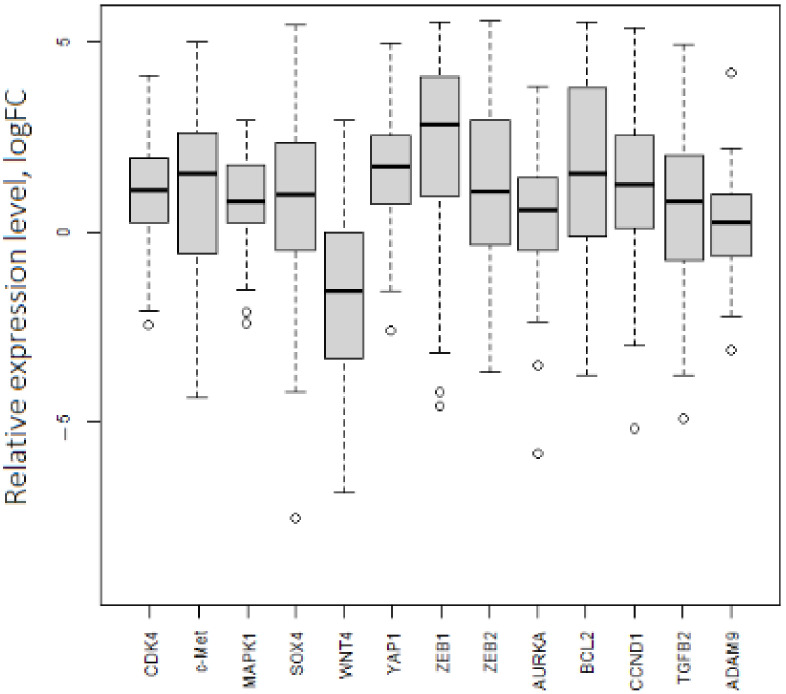
Expression levels of 13 mRNAs in OC. *p*.adj < 0.05 for each mRNA.

In the same way, we noticed a difference between expression values based on the TCGA data analysis and the obtained experimental results for mRNAs (Table 1, Figure 4). WNT4 had decreased expression (logFC = −1.6), and the expression of seven mRNAs (CDK4, MAPK1, TGFB, ZEB2, AURKA, SOX4, and ADAM9) in tumor samples was not notably different from that in the group of normal tissues. The outlying points with low expression levels were related to both the initial and late stages of OC. Expression levels of ZEB1, YAP1, CCND1, and BCL2 were consistent with the database analysis and demonstrated significantly high values in tumor tissues (logFC ≥ 1.5).

The calculation of Spearman’s coefficient for three types of pairs (miRNA–lncRNA, miRNA–mRNA, and lncRNA–mRNA) evidenced that expression levels of all five selected tumor-suppressive miRNAs were negatively correlated with those of the mRNAs and lncRNAs.

**Figure 5:**
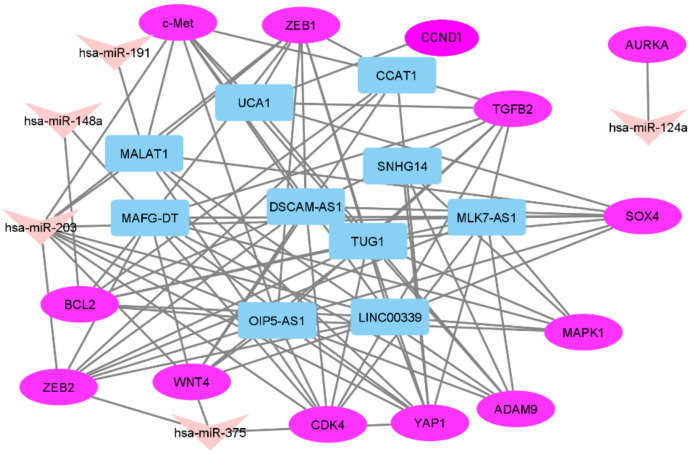
The network between three types of RNAs validated in a set of paired OC samples.

Altogether, we identified 16 triplets with significant correlation coefficients between expression levels of RNAs (Table 2). Based on Spearman’s coefficients, miR-191, miR-124a, and miR-375 did not form triplets in our sample set. The miR-191 level had a moderate correlation with the expression of only one lncRNA, MALAT1. MiR-124a and miR-375 levels correlated only with the expression of mRNAs: AURKA in the case of miR-124a and WNT4, YAP1, and ZEB2 in the case of miR-375. MiR-124a–AURKA appeared to be the most separate pair, its members did not form any other connections in the network built by us (Figure 5). Connections in the network had moderate strength of correlation in the range from −0.40 to −0.59.

We revealed only one triplet with miR-148a, which was moderately connected with MALAT1 and ADAM9 (Table 2). LncRNA–mRNA communication in this triplet was also moderate.

Fifteen triplets were formed in conjunction with miR-203a (Table 2). In all these triplets, the miR-203a level had a moderate negative correlation with the expression of both lncRNAs and mRNAs. At the same time, we observed varied positive correlations between the expression of lncRNAs and mRNAs with the highest correlation of OIP5-AS1 level with ZEB2 level.

However, the analysis of the localization of miR-203 binding sites showed that binding is possible only with two out of four correlated lncRNAs (OIP5-AS1 and MLK7-AS1), and that only c-MET, ZEB1, and ZEB2 had a complementary sequence with miR-203 among six correlated mRNAs. MiR-203 had more common nucleotides in the seed site with c-MET and ZEB2 sequences (Table 3).

### 2.3. Experimental Microarray-Based Expression Analysis on Clinical Samples

Affymetrix Human Transcriptome analysis of additional clinical samples revealed 512 DE genes, including 107 lncRNAs and 405 mRNAs. Of these, 121 DE genes were common for in silico TCGA-OV and in vitro analyses (Supplementary Table S7). The trends in the pathway dysregulation also coincided in both cohorts. Thus, the in vitro pathway analysis confirmed that oxidative stress response, cell adhesion, epithelial to mesenchymal transition, and proliferation-related pathways were among significantly dysregulated mechanisms with upregulated genes in OC patients (Supplementary Table S8).

### 2.4. Experimental Validation of the Interactions between miRNAs and mRNAs on Cell Lines

**Figure 6:**
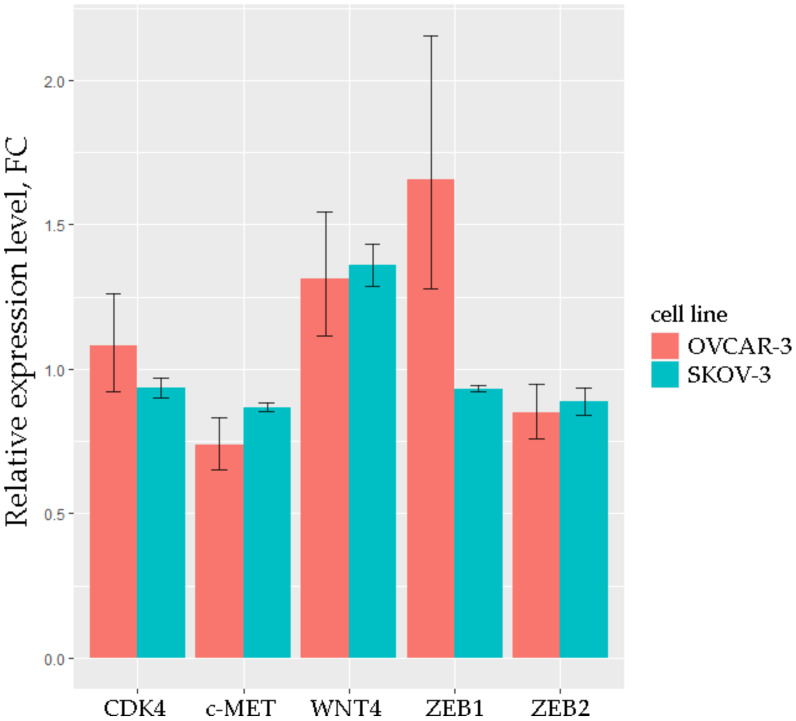
Relative expression levels of 5 mRNAs in OVCAR-3 and SKOV-3 cell lines transfected with the miR-203a mimic. *p*.adj < 0.05 for each mRNA, the results are based on three technical replicates.

To validate possible interactions between selected miRNAs and mRNAs, we conducted the transfection of OVCAR-3 and SKOV-3 cell lines by duplexes mimicking miR-203a, which was the miRNA with the greatest number of triplets. In the expression analysis, we considered the miR-203a-3p isoform because, according to miRBase, it is the most widespread mature sequence of miR-203a. The results of qRT-PCR analysis are shown in Figure 6.

The expression level of CDK4, which does not have binding sites for miR-203, was very close to that of the mock control in both transfected cell lines. The level of WNT4, which also does not have binding sites for miR-203, was increased in OVCAR-3 and SKOV-3, apparently due to additional influences. A similar increase was observed for ZEB1 in OVCAR-3. The levels of c-MET and ZEB2 (which have the best potential binding sites for miR-203, Table 3) were 15–30% lower in transfected OVCAR-3 and SKOV-3 than in mock, which is consistent with the qRT-PCR data obtained on clinical samples.

## 3. Discussion

Near 90% of all malignant ovarian tumors are epithelial tumors, wherein serous ovarian adenocarcinoma has the highest number of negative outcomes [20,21]. As a rule, this type is often associated with metastasis and loss of sensitivity to drugs [22]. Previously, our group identified a connection between the hypermethylation of a number of tumor-suppressive miRNA genes with the pathogenesis and progression of serous ovarian adenocarcinoma [12,23]. In this work, we focused on interactome changes caused by the misregulation of these miRNAs. MiRNAs are distinguished by their multitargeting effect. Each miRNA can be involved in the regulation of up to 200 mRNAs or lncRNAs [24], and vice versa, one mRNA, as well as one lncRNA, is usually a target for many miRNAs.

In the present study, the gene set enrichment analysis of DE genes indicated that the significantly dysregulated pathways were related to processes of proliferation and inflammation (Figure 1). After the evaluation of Spearman’s correlation coefficients between expression levels for lncRNA–mRNA pairs, we gave priority to the pairs involved in the processes listed above. We assumed that the aberrations in the signaling pathways, which we revealed by the GSEA, represent the initial steps of metastatic cascades: local invasion and migration, angiogenesis, epithelial–mesenchymal transition (EMT), and intravasation. Notably, EMT in association with inflammation correlates with stages III and IV of cancer progression [25].

EMT stimulates immobile epithelial cells to become mobile and increases their capacity for invasion. Various growth factors (TGF-β, PDGF, FGFR) and a number of transcription factors, mainly the ZEB, SNAIL, and TWIST families, promote EMT [26,27]. As a result, metastatic cascade coordinates biological events such as local cellular invasion and allows cancer cells to leave the primary focus, develop new blood vessels, migrate and penetrate the microenvironment, and perform intravasation and extravasation, survive in the circulation, and, finally, colonize distant organs [28]. OC shows especially high metastatic and invasive potential, firstly colonizing the peritoneum with ascites formation [29]. The predominance of intraperitoneal metastasis makes the progression of OC the most aggressive among various epithelial cancers. As a consequence, it necessitates an in-depth study of the mechanisms of the spread of this tumor, the knowledge of which could become the basis for the development of new drugs that purposefully affect the processes of metastasis and invasion.

In the experimental part of this study, we analyzed expression levels of 13 mRNAs, 10 lncRNAs, and 5 miRNAs related to EMT in a representative set of primary ovarian tumors and matched normal tissues. All miRNAs were downregulated, meanwhile, the expression values for mRNAs and lncRNAs differed from those in in silico evaluation in available datasets. ZEB1, BCL2, and YAP1 among mRNAs and MLK7-AS1, LINC00339, and OIP5-AS1 among lncRNAs had a significant increase in expression level as expected. The decreased values and values with a null difference could be a result of the sample size and composition and/or data retrieval methods (Illumina sequencing or qPCR) and control samples (pooled norm or matched norm for each tumor sample) and require further analysis. Only two miRNAs formed triplets: miR-148a and miR-203a, and miR-203a had a number of connections with lncRNAs and mRNAs. Several studies demonstrated that miR-203a functioned as a tumor suppressor in ovarian cancer, whereas inhibiting this miRNA promoted tumor growth, migration, and invasion in a xenograft mouse model [30,31]. Moreover, immunostaining of tumor sections evidenced that miR-203a inhibits EMT in vivo [30].

Notably, in our recent study [23] on a set of 102 primary tumors and 30 peritoneal metastases, the hypermethylation of miR-203a showed the most statistically significant association with metastasis (*p* < 10^−4^, FDR = 0.01). The strongest decrease in miR-203a expression in primary tumors from patients with metastases corresponded to a sharp appearance of hypermethylation of the *MIR203A* gene in primary tumors exclusively from patients with metastases. Here, we estimated all miRNA–mRNA, miRNA–lncRNA, and lncRNA–mRNA connections in which miR-203a was involved or associated (Table 2). Using the sequencing data on the localization of miR-203a binding sites (TargetScan v.7.2 and DIANA-TarBase v.8), we found that two lncRNAs, namely, OIP5-AS1 and MLK7-AS1, contained binding sites, and the same was observed for three mRNAs—c-MET, ZEB1, and ZEB2, among which c-MET and ZEB2 had the best characteristics (8-mer) for binding sites (Table 3).

The regulatory mechanisms of the lncRNAs are as yet insufficiently studied. There are three main directions of lncRNA impact: direct modification and structuring of chromatin into domains, loops, supercoils, and chromosomes; binding to proteins; and interactions with RNAs [32]. In the last case, lncRNAs can act as sponges in regulating mRNA expression positively by targeting miRNAs. In this study, we investigated hypermethylated low-expressed miRNAs. Thus, we assumed that both other elements in the triplet, i.e., lncRNA and mRNA, should be upregulated. Indeed, the strongest positive correlation was noted for OIP5-AS1–ZEB2. Recent studies revealed a direct negative feedback loop between miR-203a and ZEB2 participating in tumor stemness and chemotherapy resistance, in which increased miR-203a expression sensitized cancer cells to cisplatin in vitro [33,34].

c-MET is a mesenchymal–epithelial transition factor involved in the progression of tumors of various sites by stimulating proliferation, motility, invasiveness, morphogenesis, angiogenesis, and EMT [35]. Since the discovery of the oncogenic functions of c-MET, many efforts have been made to develop anticancer drugs that target this oncoprotein [36]. In many recently discovered regulatory axes as lncRNA–miRNA–mRNA, c-MET acts as a target. For example, the lncRNA HOTAIR/miR-613/c-MET signaling axis is involved in modulating the expression of EMT-specific proteins, apoptosis, and retinoblastoma cells’ viability [37]. A dozen miRNAs (miR-1, miR-148a-3p, etc.) were identified that, acting on the c-MET mRNA, suppressed EMT and OC progression [38,39]. Moreover, a regulatory axis with the participation of circular RNA Circ0004390–miR-198–c-MET has been discovered, which reduces the survival rate of patients with OC [40]. However, the regulation of c-MET involving any lncRNA in OC was not reported, and the interaction of c-MET with miR-203a in OC was revealed by us for the first time. All these data along with our results suggest the existence of coregulation between players in triplets such as OIP5-AS1–miR-203a–ZEB2 and OIP5-AS1–miR-203a–c-MET, which is imbalanced by cancer development and may stimulate EMT and metastasis of OC.

In vitro analysis by the array-based method revealed 121 DE genes, which were common with samples from the TCGA database. Furthermore, the pathway analysis confirmed the general trends in the dysregulation of molecular mechanisms. For validation of the selected RNAs in the cell model we chose two cell lines: OVCAR-3 and SKOV-3. SKOV-3 is a cell line with epithelial morphology that was isolated from the primary tumors of patient with ovarian adenocarcinoma (HTB-77). OVCAR-3 comprises epithelial cells isolated from the malignant ascites of a patient with progressive adenocarcinoma of the ovary (HTB-161). Notably, the OVCAR-3 cell line originates from metastases with ascitic fluid of a progressive adenocarcinoma. These differences between cell lines may explain the diverse effects of the transfection of miR-203a mimic on the mRNA of the studied genes. Nevertheless, the c-MET and ZEB2 levels were decreased in both OVCAR-3 and SKOV-3, which indicates the direct binding of miR-203a to these mRNAs.

Once a malignant neoplasm has become metastatic, the tumor cells are found in the peripheral blood. Such circulating cells are the source of tumor RNA, which can be profiled to detect imbalanced triplets. Though the RNA concentration in plasma is significantly lower compared to that in tumor tissue samples, we suggest that the shift in the lncRNA–miRNA–mRNA interactome can still be determined.

## 4. Materials and Methods

### 4.1. Bioinformatic Analysis

**Figure 7:**
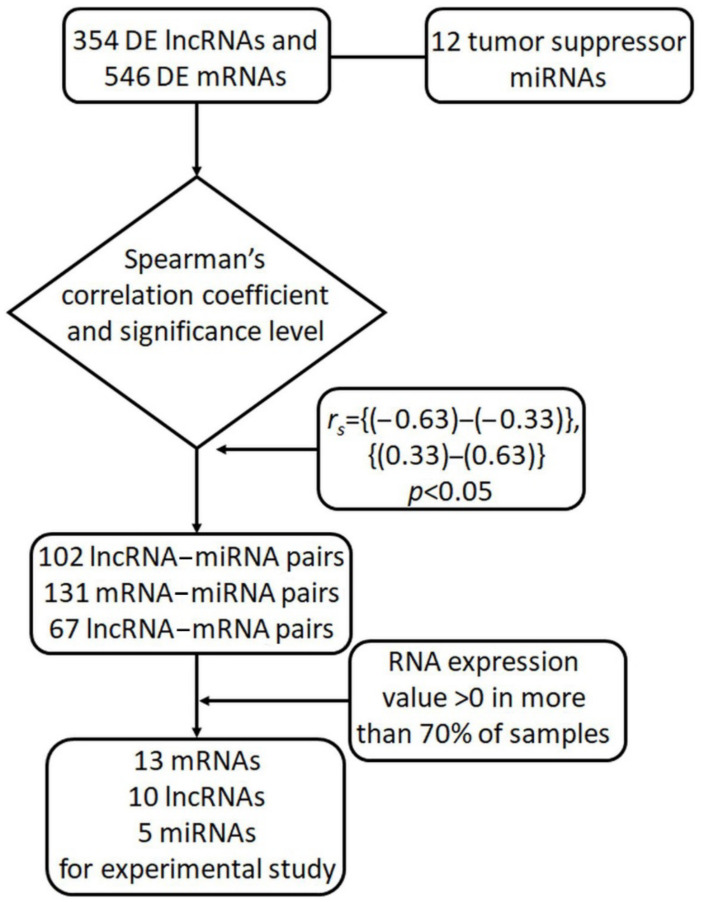
The workflow of bioinformatic analysis for identification of lncRNA–miRNA–mRNA triplets.

We extracted counts of reads from the TCGA-OV project, study accession phs000178 (https://portal.gdc.cancer.gov/projects/TCGA-OV, accessed on 27 November 2021) for a tumor group and from the GTEx portal (https://gtexportal.org/, accessed on 27 November 2021) for a normal group. The counts in datasets were normalized by FPKM (fragments per kilobase per million) values. Using the removeBatchEffect procedure from the limma package in R studio, we eliminated systematic errors that occur during the sequence alignment stage. The limma-voom function was applied for DE analysis of lncRNAs and mRNAs: only the expression variations across samples (log2IQR) > 0.5 with an adjusted *p*-value < 0.05 were considered significant. The false discovery rate (FDR) was controlled at the 0.01 threshold. We used TargetScan v.7.2, DIANA-TarBase v.8, and mirPath v.3 for sequence target prediction of the above-mentioned 12 miRNAs. The bioinformatic pipeline is presented in Figure 7.

For 546 DE mRNAs, we performed gene set enrichment analysis with the fgsea R-package and database of Kyoto Encyclopedia of Genes and Genomes (KEGG), with *p*.adj ≤ 0.05.

We calculated Spearman’s correlation coefficients between expression levels for miRNA–lncRNA and miRNA–mRNA pairs. The target prediction for miRNAs was verified by TargetScan v.7.2, DIANA-TarBase v.8, and mirPath v.3. Then, the lncRNA and mRNA levels were analyzed to evaluate the regulatory relationships between these three RNA types in each triplet. The Spearman’s correlation coefficient and *p*-level of correlation were processed using the R studio cor.test function. For miRNA–lncRNA and miRNA–mRNA pairs, only negative correlations whose significance level was less than the specified threshold (*p*.adj < 0.05) were selected. The cutoff values of the correlation coefficient for the miRNA–lncRNA and miRNA–mRNA pairs were −0.33, while for lncRNA–mRNA, it was 0.4.

### 4.2. Clinical Data

The study included paired samples of tumor and unchanged ovarian tissues from 46 patients, which were collected and morphologically characterized at the N.N. Blokhin National Medical Research Center of Oncology, Russia, Moscow. Most samples (71%, 33/46) were serous ovarian adenocarcinomas. Only the samples containing 70–80% or more tumor cells were used in the study. Patients did not receive chemotherapy prior to surgery. All samples were classified according to the TNM classification and histologically verified based on the WHO classification criteria. Clinical and morphological data are shown in Table 4. The study was performed in accordance with the Declaration of Helsinki. The samples were collected under the guideline issued by the Ethics Committee of the N.N. Blokhin National Medical Research Center of Oncology. Tissue samples were stored at −70 °C.

### 4.3. RNA Isolation

Samples up to 5 mm^3^ in volume obtained during biopsy or surgery were homogenized by an Ultra-Turrax T 10 basic disperser (IKA, Staufen, Germany). Total RNA was isolated according to the guanidine–thiocyanate–phenol–chloroform extraction protocol with modifications as given previously [41]. The total RNA concentration was determined spectrophotometrically at 260 nm by the optical density on the NanoDrop ND-1000 spectrophotometer (Thermo Fisher Scientific, Waltham, MA, USA). We estimated RNA quality using the absorbance coefficients A260/230 and A260/280. The preservation of RNA was determined by the ratio of the intensities of the 28S rRNA and 18S rRNA bands during electrophoresis in a 1% denaturing agarose gel. All RNA samples were treated with RNase-free DNase prior to use.

### 4.4. Reverse Transcription and qPCR

Reverse transcription of RNA was performed using MMLV reverse transcriptase (Evrogen, Russia) and random hexanucleotide primers. Each setting of the reverse transcription reaction included negative controls containing no RNA.

We estimated expression levels of 10 lncRNAs, 13 mRNAs, and 5 miRNAs by qPCR on a CFX96 thermal cycler (Bio-Rad, USA). A SYBR Green intercalating dye, qPCRmix-HS SYBR (Evrogen), was added to the PCR mixture. The resulting mix was dispensed into a 96-well plate. The total volume of the PCR reaction was 20 μL. Conditions for amplification of DNA fragments were: 95 °C for 20 s—1 cycle; 95 °C for 1 s, 60–64 °C for 20 s—40 cycles. For lncRNAs and mRNAs, we used the primers presented in Supplementary Table S6; the *B2M* gene was a reference gene.

The expression levels of miRNAs were analyzed using TaqMan MicroRNA Assays (Applied Biosystems, USA): miR-124-3p (Assay ID: 001182), miR-148-3p (Assay ID: 00470), miR-191-5p (Assay ID: 002299), miR-203a (Assay ID: 000507), and miR-375 (Assay ID: 000564). RNU48 (Assay ID: 001006) and RNU6 (Assay ID: 001093) expression levels were used as references. The presence and length of the cDNA were checked by electrophoresis of PCR products in 1% agarose gel with an EtBr intercalating dye.

### 4.5. Cell Lines

Two ovarian cell lines were used. SKOV-3 is a cell line with epithelial morphology that was isolated from the ovary of a 64-year-old White female with ovarian adenocarcinoma (HTB-77)*,* and OVCAR-3 comprises epithelial cells isolated from the malignant ascites of a patient with progressive adenocarcinoma of the ovary (HTB-161).

Cell lines were purchased from the CLS Cell Lines Service GmbH (Eppelheim, Germany). Cell line characteristics, including karyotype analysis data, were provided by the vendor. The identity of the cell line was not authenticated further. The cells were cultured in Dulbecco’s modified Eagle’s medium (DMEM; Life Technologies, Carlsbad, CA, USA) supplemented with 10% (*v*/*v*) fetal bovine serum (FBS) (Life Technologies), 50 µg/mL gentamicin (Life Technologies), 4 mM L-glutamine, and 4.5 g/L D-glucose, in a humidified atmosphere containing 5% CO_2_ in an incubator (Sanyo, Japan) at 37 °C. The cells were subcultured at confluence by treatment with 0.05% trypsin and 0.02% EDTA in phosphate-buffered saline (PBS).

### 4.6. RNA Duplexes and Transfection

Synthetic miRNAs were designed to mimic mature endogenous miRNAs. Sequences of hsa-miR-203-5p and its antisense strand were identical to the strands of RNA duplexes used. We introduced 3′-overhangs and the thermodynamic destabilization of the relevant seed-containing end of the duplexes to facilitate activation of the sense strand. miRNA duplexes were synthesized (DNA-Sintez, Russia), resuspended at 200 μM, annealed by heating to 95 °C, and then slowly cooled to 37 °C. For transfection, 0.4 million cells were plated per well in a six-well plate supplied with 2 mL of medium (DMEM + 10% FBS). After 24 h of culture (at 50% to 80% confluence), the cells were supplied with fresh medium and transfected with an RNA duplex at a final concentration of 40 nM using Lipofectamine 2000 (Thermo Fisher Scientific) and Opti-MEM (Thermo Fisher Scientific) following the manufacturer’s protocol. After 24 h, the cells were harvested and analyzed. Transfection of cel–miR-67-3p RNA duplexes exhibiting low homology with human miRNA sequences was used as a specificity control, whereas assessment of expression for hsa–miR-1-3p RNA duplexes together with their target twinfilin actin binding protein 1 (TWF1) was used as a positive control.

### 4.7. Affymetrix Whole-Transcriptome Gene Expression Analysis

A Human Transcriptome Array 2.0 System (Thermo Fisher Scientific) was used for gene expression analysis. Biotinylated sense-strand DNA targets were prepared from 500 ng of total RNA using the GeneChip WT PLUS Reagent kit (Ambion). Hybridization, labeling, and washing were performed using the GeneChip Hybridization Wash and Stain Kit (Thermo Fisher Scientific) using Fluidics Station 450 (Affymetrix). The arrays were scanned using a Gene Scan 7G (Affymetrix) system. Standard Affymetrix quality control was conducted using the Expression Console Software (Affymetrix). Gene set enrichment analysis (GSEA) was applied for interpreting genome-wide expression profiles using GenePattern (https://cloud.genepattern.org, accessed on 23 March 2022).

Clinical samples used in this study were taken from two women with high-grade serous ovarian carcinoma, aged 70 and 43 years, both characterized by T3cN0M0 and stage IIIC. Samples taken from both women contained peritoneal metastases and fallopian tube tissue as normal tissue controls [42]. The RNA isolated from these samples was tested for integrity on an Agilent 2100 Bioanalyzer. Sample collection was performed using the Agilent RNA 6000 Nano Assay Protocol. The RIN (The RNA integrity number) of the samples used was 5.4–6.2, which is close to the required value [43].

### 4.8. Statistical Methods

R studio was used to calculate median and interquartile range and to build the correlation matrix. The significance of differences was obtained by the nonparametric Mann–Whitney U test. The Spearman’s correlation coefficient (*r_s_*) was calculated using the same parameters as for in silico analysis. Differences were considered statistically significant at *p*.adj < 0.05.

## 5. Conclusions

Highly expressed miRNAs have been suggested to be more significant for cancer diagnostics; however, poorly expressed miRNAs may also be markers for carcinogenesis. In the present study, we analyzed interactions of three types of RNAs according to the model of competitive endogenous RNA, in which miRNA is bound alternatively or competitively either to mRNA or lncRNA. In this case, miRNA acts as a mediator between both lncRNA and mRNA, while lncRNA and mRNA may compete with each other for miRNA binding. We showed for the first time that the downregulated tumor-suppressive miR-148a and miR-203a induced an imbalance in their RNA interactome. In experimental validation, we focused on EMT-related lncRNAs and mRNAs. Two triplets attracted attention, OIP5-AS1–miR-203a–ZEB2 and OIP5-AS1–miR-203a–c-MET, due to the strong correlation between expression levels of lncRNA, miRNA, and mRNA players and due to the presence of miR-203a binding sites in OIP5-AS1, ZEB2, and c-MET. The interaction of miR-203a with ZEB2 was revealed for the first time in OC, and with c-MET for the first time in oncogenesis. The possibility of miR-203a direct binding to ZEB2 and c-MET was shown by transfection of the OVCAR-3 and SKOV-3 cell lines with the miR-203a mimic. The high tissue specificity of such interactions benefits the expression profiling for diagnostics and prediction of a number of cancers, including OC.

## Figures and Tables

**Table 1:** Characteristics of the selected RNAs.

RNA	Chromosome (Position) *	Localization in a Cell **	LogFC (TCGA)	LogFC ((qPCR)
**lncRNAs**				
MALAT1	Chr11 (65,497,738–65,506,516)	Nucleus	5.0	1.1
MAFG-DT	Chr17 (81,927,829–81,930,753)	Extracellular space	2.9	0.8
OIP5-AS1	Chr15 (41,282,697–41,313,338)	Nucleus	5.0	3.3
MLK7-AS1	Chr2 (173,197,712–173,282,036)	No information	1.5	2.5
LINC00339	Chr1 (22,025,191–22,031,224)	Extracellular space, cytosol, and nucleus	1.5	2.5
TUG1	Chr22 (30,969,211–30,979,395)	Extracellular space, cytosol, and nucleus	38.8	2.1
UCA1	Chr19 (15,828,947–15,836,321)	Extracellular space, cytosol, and nucleus	1.6	1.4
SNHG14	Chr15 (24,823,608–25,419,462)	Nucleus	2.3	−1.1
CCAT1	Chr8 (127,207,382–127,219,268)	Nucleus	11.6	1.3
DSCAM-AS1	Chr21 (40,383,083–40,385,358)	Extracellular space, cytosol, and nucleus	27.7	1.4
**miRNAs**				
miR-148a	Chr7 (25,949,919–25,949,986)	Extracellular space and vesicles	N/A	−4.0
miR-191	Chr3 (49,020,618–49,020,709)	Extracellular space and vesicles	N/A	−3.8
miR-203a	Chr14 (104,117,405–104,117,514)	Nucleus	N/A	−2.1
miR-124a	Chr8 (9,903,388–9,903,472)	Cytoplasm and extracellular exosomes	N/A	−3.4
miR-375	Chr2 (219,001,645–219,001,708)	Extracellular space	N/A	−2.5
**mRNAs**				
AURKA	Chr20 (56,369,390–56,392,308)	Nucleus	1.8	1.2
BCL2	Chr18 (63,123,346–63,320,280)	Nucleus	3.2	1.8
CDK4	Chr12 (57,747,727–57,752,310)	Nucleus	2.1	1.3
c-MET	Chr7 (116,672,196–116,798,386)	Extracellular space	3.2	1.6
WNT4	Chr1 (22,117,308–22,143,981)	Extracellular space	2.7	−1.6
YAP1	Chr11 (102,109,957–102,233,424)	Nucleus and cytosol	2.2	2.0
ZEB1	Chr10 (31,318,417–31,529,804)	Nucleus and cytosol	2.1	2.9
ZEB2	Chr2 (144,384,081–144,520,119)	Nucleus and cytosol	1.7	1.1
CCND1	Chr11 (69,641,156–69,654,474)	Nucleus and cytosol	3.2	1.5
ADAM9	Chr8 (38,996,767–39,105,261)	Extracellular space	8.0	0.3
SOX4	Chr6 (21,593,751–21,598,619)	Nucleus	1.8	1.2
TGFB	Chr19 (41,330,323–41,353,922)	Extracellular space, cytosol, and nucleus	1.8	0.9
MAPK1	Chr22 (21,759,657–21,867,680)	Extracellular space, cytosol, and nucleus	1.9	0.9

LogFC—binary logarithm of expression level fold change (tumor vs. normal). * The genome coordinates are specified according to the hg38 genome assembly. ** The localization of the RNAs is included according to the UniProt database (https://www.uniprot.org/, accessed on 9 December 2021).

**Table 2:** Spearman’s correlation coefficients between expression levels for different pairs of RNAs.

Type of RNA		*r_s_* *
**miRNA**	**lncRNA**	
miR-148a	MAFG-DT	−0.37
miR-203a	MALAT1	−0.42
	OIP5-AS1	−0.42
	MLK7-AS1	−0.41
	LINC00339	−0.39
**miRNA**	**mRNA**	
miR-148a	BCL2	−0.44
miR-203a	CDK4	−0.54
	c-MET	−0.42
	ZEB1	−0.34
	ZEB2	−0.41
	WNT4	−0.41
	YAP1	−0.53
**lncRNA**	**mRNA**	
MAFG-DT	BCL2	0.45
MALAT1	CDK4	0.52
	c-MET	0.39
	ZEB1	0.38
OIP5-AS1	CDK4	0.53
	c-MET	0.37
	WNT4	0.56
	YAP1	0.61
	ZEB1	0.57
	ZEB2	0.65
MLK7-AS1	CDK4	0.40
	YAP1	0.51
	ZEB2	0.49
LINC00339	CDK4	0.53
	YAP1	0.55
	ZEB1	0.43

* *p*.adj < 0.05 for all Spearman’s correlation coefficients.

**Table 3:** Data on the possibility of miR-203 binding to the selected lncRNAs and mRNAs.

RNA	Sequence (GRCh37/hg19)	Binding Site
	**lncRNAs**	
MALAT1	no binding site	-
OIP5-AS1	chr15: 41,593,028–41,593,127	7-mer-m8
MLK7-AS1	chr2: 174,080,356–174,080,455	7-mer-m8
LINC00339	no binding site	-
	**mRNAs**	
CDK4	no binding site	-
c-MET	chr7: 116,415,099–116,415,198	8-mer
WNT4	no binding site	-
YAP1	no binding site	-
ZEB1	chr10: 31,817,289–31,817,388	7-mer-m8
ZEB2	chr2: 145,184,392–145,184,491	8-mer

The data are presented according to TargetScan v.7.2 and DIANA-TarBase v.8.

**Table 4:** Clinical and histological characteristics of OC patients and tissue samples.

Clinical Characteristics		N (%)
Age, years	<40	7 (15)
	40–60	22 (48)
	>60	17 (37)
Histological type	Borderline serous adenocarcinoma	3 (6)
	Serous adenocarcinoma	33 (72)
	Endometrioid adenocarcinoma	5 (12)
	Clear cell adenocarcinoma	1 (2)
	Mixed epithelial tumors	2 (4)
	Undifferentiated carcinoma	2 (4)
Stage	I	12 (26)
	II	10 (22)
	III	22 (48)
	IV	2 (4)
Primary tumor site and size	T1	12 (26)
	T2	10 (22)
	T3	24 (52)
Lymph node	Nx	16 (34)
	N0	30 (65)
	N1	10 (21)
Peritoneal metastases	Absent	32 (69)
	Present	14 (31)

## Data Availability

The data presented in this study are available on reasonable request from the corresponding author.

## Supplementary Materials

The following are available online at https://www.mdpi.com/article/10.3390/biomedicines10040824/s1, Table S1: Differential expression of lncRNAs in a group of OC patients and healthy individuals; Table S2: Differential expression of mRNAs in a group of OC patients and healthy individuals; Table S3: Spearman’s correlation coefficients between miRNA and lncRNA levels; Table S4: Spearman’s correlation coefficients between miRNA and mRNA levels; Table S5: Spearman’s correlation coefficients between mRNA and lncRNA levels; Table S6: Primer sequences and temperature conditions; Table S7: Differential expression of mRNAs in a group of OC patients compared to a group of healthy individuals evaluated by array-based in vitro analysis; Table S8: Dysregulated pathways in OC according to the results of array-based analysis.
