# Supplementary material for: Dysregulation of lncRNA–miRNA–mRNA Interactome as a Marker of Metastatic Process in Ovarian Cancer
Source: Biomedicines. 2022 Mar 31;10(4):824. doi: 10.3390/biomedicines10040824 (PMC9031843; doi:10.3390/biomedicines10040824)
Supplement: Supplementary file 1 [file biomedicines-10-00824-s001.zip › Supplementary Table S6.pdf]

Supplementary Table S6. Primer sequences and temperature conditions.

RNA	Primer sequences	T, °C
CCAT1	F: CTCCAATGGTGAGGAGGTTTAG	62
	R: GGCTAGTTAGAGCCGAGATTTG	
SNHG14	F: CCTCCTCCCTCAACCAAATATC	62
	R: GCCAGAACTACAGTGTCAGTAA	
MALAT1	F: GGCTCTTCCTTCTGTTCTAGTG	62
	R: CCTCCCATCCCTCCAAATTC	
MAFG-DT	F: GAGACCAGCATTGGTAACATAGA	60
	R: CTCCTGAATAGCTGGGACAAC	
OIP5-AS1	F: GGAGAGGAACTAACCGAACATT	61
	R: GCGCCTAACACTTTGCTTTAG	
MLK7-AS1	F: GCTGGGAGAGGTAAGTTGAAG	63
	R: AACTCCATCTCCTGCGTTTAG	
LINC00339	F: CCCATGTCTGGATCTCTCTTTG	62
	R: AGCAGGCTGATTGTCCTAAC	
TUG1	F: GTGCAGAAGCCCAGAGTAAA	62
	R: CCACGGTGGTAAAGGAAGATAG	
UCA1	F: GACCCTACCCGGTCATTTATAG	63
	R: CTGATGGGCATGGCTTTATTC	
DSCAM-AS1	F: CAGAGAATGCTGAGAGCAGAG	63
	R: TCCCAAAGTGCTGGGATTAC	
c-MET	F: AGCGTCAACAGAGGGACCT	62
	R: GCAGTGAACCTCCGACTGTATG	
TGFB2	F: TGCAGAACCCAAAAGCCAGAGTG	60
	R: TACAAAAGTGCAGCAGGGACAGTG	
B2M	F: TGACTTTGTCACAGCCCAAGATAG	64
	R: CAAATGCGGCATCTTCAAACCTC	
WNT4	F: AGGAGGAGACGTGCGAGAAA	62
	R: CGAGTCCATGACTTCCAGGT	
MAPK1	F: TCACACAGGGTTCCTGACAGA	61
	R: ATGCAGCCTACAGACCAAATATC	
AURKA	F: TGGGTGGTCAGTACATGCTC	61
	R: TGCATCCGACCTTCAATCATTTC	
BCL2	F: GGTGGGGTCATGTGTGTGG	62
	R: CGGTTCAGGTACTCAGTCATCC	
CDK4	F: TCAGCACAGTTCGTGAGGTG	62
	R: GTCCATCAGCCGGACAACAT	

YAP1	F: TAGCCCTGCGTAGCCAGTTA	62
	R: TCATGCTTAGTCCACTGTCTGT	
ZEB1	F: GAGTTGGAAAGGGCCTACTG	62
	R: GTTGTTATGGTGGGCATGTATTT	
ZEB2	F: CAAGAGGCGCAAACAAGCC	63
	R: CAGCTACGCCTTCTCGGTCT	
CCND1	F: TGGAGCCCGTGAAAAAGAGC	61
	R: TCTCCTTCATCTTAGAGGCCAC	
ADAM9	F: TCCATTGCTCTTAGCGACTGT	60
	R: GGGGTTCAATCCCATAACTCG	
SOX4	F: AGCGACAAGATCCCTTTCATTC	62
	R: CGTTGCCGGACTTCACCTT	
